# Evaluating Glial Fibrillary Acidic Protein and Neurofilament Light as Potential Biomarkers for Spinocerebellar Ataxia 7

**DOI:** 10.3390/ijms26115070

**Published:** 2025-05-24

**Authors:** Rana Hanna Al-Shaikh, Karen Jansen-West, Audrey Strongosky, Zoe Parrales, Judith A. Dunmore, Yuping Song, Tania F. Gendron, Juan C. Guevara, Helio A. G. Teive, Jarosław Dulski, Jarosław Sławek, Leonard Petrucelli, Zbigniew K. Wszolek, Mercedes Prudencio

**Affiliations:** 1 Department of Neuroscience, Mayo Clinic, Jacksonville, FL 32224, USA; 2 Department of Neurology, Mayo Clinic, Jacksonville, FL 32224, USA; 3 Neuroscience Graduate Program, Mayo Graduate School, Mayo Clinic College of Medicine, Jacksonville, FL 32224, USA; 4 Department of Science, University of Barcelona, 08007 Barcelona, Spain; 5 Movement Disorders Unit, Neurology Service, Internal Medicine Department, Hospital de Clínicas, Federal University of Paraná, Curitiba 80060-90, PR, Brazil; 6 Division of Neurological and Psychiatric Nursing, Medical University of Gdańsk, 80-211 Gdansk, Poland; 7 Neurology Department, St. Adalbert Hospital, Copernicus PL Ltd., 80-462 Gdansk, Poland

**Keywords:** ataxia, NfL, GFAP, biomarker

## Abstract

Spinocerebellar ataxia type 7 (SCA7), a rare form of ataxia, possesses a wide phenotypic spectrum ranging from classic ataxic symptoms to blindness, multiorgan failure, cardiomyopathy, and early death among younger age groups. Biomarkers associated with disease progression and severity could aid in disease prognostication. We evaluated the utility of glial fibrillary acidic protein (GFAP) and neurofilament light (NfL) in distinguishing patients with SCA7 from healthy controls and estimating patient prognosis. GFAP and NfL levels were measured in 23 plasma and 20 cerebrospinal fluid (CSF) samples from asymptomatic (N = 3) and symptomatic SCA7 participants (N = 10) and from healthy controls (N = 8). GFAP concentrations were elevated in the plasma (82.7 pg/mL) and CSF (9318 pg/mL) of patients with SCA7 compared to controls (plasma: 48.0 pg/mL; CSF: 89,056 pg/mL). Similarly, NfL plasma (21.6 pg/mL) and CSF (2615.0 pg/mL) levels were also significantly upregulated in SCA7 compared to controls (plasma: 8.2 pg/mL; CSF: 414.6 pg/mL). Higher levels of NfL, but not of GFAP, significantly discriminated symptomatic SCA7 patients from controls (area under de curve, AUC: 0.898, *p* = 0.0059, in plasma, and AUC: 1.0, *p* = 0.0012, in CSF). The levels of both biomarkers increased overtime, with plasma NfL levels strongly associated with a worse score in the scale for the assessment and rating of ataxia (SARA) (Spearman r: 0.8354, *p* = 0.0007; regression analysis: β: 0.021, 95% CI: 0.008–0.035, *p* = 0.0048). These findings suggest that NfL could serve as a valuable biomarker for monitoring disease progression and prognosis in SCA7 patients.

## 1. Introduction

Spinocerebellar ataxia type 7 (SCA7, OMIM#164500) is 1 of 50 types of autosomal dominant cerebellar ataxias identified to date [1,2,3]. With a prevalence of 1 to 5 in 100,000 individuals worldwide, SCAs are not as frequently encountered when compared to other movement disorders. SCA7 is extremely rare and may present in <1:100,000 individuals [4]. It is most prevalent in a few geographical regions around the globe, including Mexico, Sweden, South Africa, and Venezuela [5,6,7,8,9]. SCA7 is caused by a cytosine–adenine–guanine (CAG) polyglutamine (polyQ) repeat expansion located within exon 3 of the ataxin 7 (*ATXN7*) gene [10,11] on chromosome 3p14.1 [3]. The *ATXN7* gene encodes an 892 amino acid protein, with the N-terminal polyglutamine expansion altering the assembly of the PT3-TAFII31-GCN5L acetylase (STAGA) chromatin-modifying complex involved in transcriptional regulation [12,13]. The normal length of CAG repeats in *ATXN7* ranges from 3 to 20, indicating that *ATXN7* can only handle a limited number of repeats before it becomes pathogenic [13]. When the number of CAG repeats exceeds the normal range, it can lead to SCA7. There are two categories of pathogenic repeat expansions: reduced penetrance (34 to 36 CAG repeats), where individuals may or may not develop disease, or full penetrance (37 to 460 CAG repeats), where individuals are highly likely to develop disease [3]. Anticipation, a phenomenon where disease worsens and appears earlier in each generation of a family, is frequently observed in CAG-repeat SCAs and presents more intensely among SCA7 mutation carriers [3,14]. Due to its unstable nature, the CAG repeat expansion mutation in SCA7 expands with each successive generation, leading to a variation in disease manifestation [12]. Members of the successive generations may develop more severe symptoms and at a younger age, years prior to the development of symptoms in their parents or grandparents [15]. A unique aspect of SCA7 is its propensity to manifest across different age groups, including infants, juveniles, adolescents, and adults. Additionally, longer CAG repeats, particularly those with 100 or more repeats, are more frequently observed in younger patients with SCA7, often leading to a more severe clinical course or even death. This pattern of anticipation is notably distinct from other CAG repeat diseases and usually displays poor prognosis [3]. The clinical manifestation of SCA7 includes typical characteristics of other spinocerebellar ataxias (SCAs): gait impairment, incoordination, nystagmus, dysarthria, dysmetria, ocular motor disturbances, dysdiadochokinesia, hyperreflexia, peripheral neuropathy, and postural tremor [8]. SCA7 presents with some clinical features that are distinct from other SCAs, such as visual impairment that can progress to complete vision loss due to the gradual degeneration and dystrophy of retinal rods and cones [16,17,18]. All aforementioned traits contribute to the wide spectrum of clinical phenotypes and disease severity ranging from vision impairment during adult onset [17,19] to failure to thrive, developmental delay, multiorgan failure, and cardiomyopathy, eventually leading to death at a very early age [3,15]. Advancements in genetic analysis have facilitated the determination of a definitive diagnosis; however, predicting prognosis and survival and improving patient care are still unattainable without molecular indicators. Accordingly, in this paper, we investigate the biomarker utility of two intermediate filaments—glial fibrillary acidic protein (GFAP), a marker of astrogliosis, and neurofilament light (NfL), a marker of neuroaxonal damage and neurodegeneration. While GFAP has been associated with other neurodegenerative diseases [20,21,22,23], it has not been investigated as a biomarker for SCA7. Moreover, NfL has been detected in biofluids from patients with various neurodegenerative diseases [24,25,26,27,28,29], including SCA3, pre-symptomatic and early-onset SCA7, and other SCAs [30,31,32,33,34,35,36,37,38]. Therefore, we measured NfL and GFAP levels in the plasma and cerebrospinal fluid (CSF) of individuals with asymptomatic and symptomatic SCA7 and controls to evaluate their potential as disease biomarkers.

## 2. Results

### 2.1. Study Participants

Our cohort consists of asymptomatic (N = 3) and symptomatic SCA7 participants (N = 10) and healthy controls (N = 8), with a total of 7 females in the asymptomatic (N = 2) and symptomatic (N = 5) SCA7 groups (Table 1). SCA7 individuals originated from Mexico, Poland, Spain, and the United States. The Polish patients were previously reported [39], and our cohort contained one child with an onset of ataxia at the age of 7 years. The median age of ataxia onset in our cohort of patients with SCA7 was 30 years, with a range from 7 to 55 years of age (Table 1). Symptoms at disease onset included vision impairment (N = 3) and/or gait difficulties (N = 6), tremor (N = 1), and dysarthria (N = 1). Median disease duration from age of onset to time of first sample collection was 8.61 for the seven patients of SCA7 with CSF and 8.73 for all SCA7 patients with plasma (Table 1). The clinical rating scales utilized in our study were the scale for the assessment and rating of ataxia (SARA) [34] and gait mobility scale (GMS) [40]. The median SARA score among the symptomatic SCA7 patients was 11.5 (range: 3–31, out of 40 points) (Table 1). As for the GMS, the median score was 1, corresponding with impaired gait with no assistance required (range: 0 to 4) (Table 1). At the first visit, most patients with SCA7 also showed vision changes (N = 8) and/or abnormal reflexes (N = 8).

### 2.2. Length of ATXN7 CAG Repeat Expansion Is Associated with Age of Ataxia Onset

The association between longer CAG repeat length in *ATXN7* and earlier age of symptom onset was previously established [3,38,41,42]. We thus evaluated whether *ATXN7* CAG repeat length is associated with age of symptom onset in our cohort of patients with SCA7. The median number of CAG repeats in the symptomatic SCA7 cohort, determined through a research lab-based polymerase-chain reaction assays, was 39 CAG repeats, with a range from 34 to 43 CAG repeats. Of note, results from clinically approved genetic assays for CAG *ATXN7* repeat-length are also available for six individuals in the cohort and show a slightly higher number of repeats than the research-based laboratory assays (Table S1), where symptomatic SCA7 showed a median number of 43 *ATXN7* CAG repeats (range: 41–50). Since clinical-based assays were not available for all study participants, we used the research lab-based assay results for subsequent analyses. We observed that an earlier age of ataxia onset correlated with longer *ATXN7* CAG repeat expansions (Spearman r = −0.8598, *p* = 0.0023, N = 10; Figure S1), analogous to previously published studies [38,41].

### 2.3. GFAP Concentrations in Plasma or CSF Do Not Distinguish Patients with SCA7 from Healthy Controls

**Figure 1:**
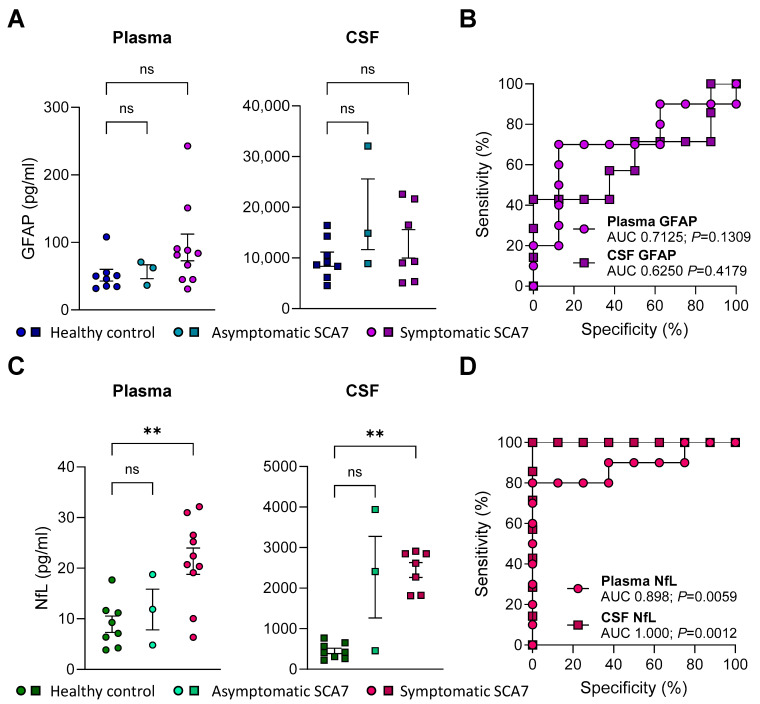
GFAP and NfL levels in the plasma and CSF of SCA7 patients demonstrate plasma NfL levels discriminate symptomatic SCA7 patients the best from the healthy controls. (**A**,**C**) GFAP (**A**) and NfL (**C**) levels were measured in plasma (left, circles) and CSF (right, squares) using commercially developed kits (see Section 4), in symptomatic and pre-symptomatic SCA7 individuals and healthy controls. Statistical differences were assessed by Kruskal–Wallis followed by Dunn’s multiple comparison test. ** *p* < 0.005, ns: not significant. (**B**,**D**) Area under the receiver operating curve (AUC) analyses for GFAP (**B**) and NfL (**D**) levels in plasma and CSF to assess their ability to distinguish symptomatic SCA7 patients from the healthy controls.

Based on emerging evidence demonstrating increased GFAP concentrations in biofluids from individuals with neurodegenerative diseases [20,21,22,23], we evaluated whether plasma or CSF GFAP is elevated in SCA7 patients compared to the controls. Trends of higher plasma and CSF GFAP levels were observed in symptomatic SCA7 patients compared to the healthy controls (Figure 1A), where the median plasma and CSF GFAP concentrations were 82.7 and 9318 pg/mL, respectively, in SCA7 compared to 48 and 8906 pg/mL in the plasma and CSF of controls (Table 1). Of note, we also included the evaluation of three asymptomatic *ATXN7* mutation carriers and, while these numbers are too low to establish meaningful outcomes, we observed a similar trend of higher GFAP in the CSF of asymptomatic SCA7 (148,801 pg/mL) compared to the controls (8906 pg/mL; Figure 1A and Table 1). We additionally performed a receiver operator characteristic (ROC) curve analysis to evaluate the ability of GFAP levels to discriminate between symptomatic SCA7 individuals and the controls. The area under the curve (AUC) values were higher when using GFAP concentrations in plasma (AUC: 0.7125, *p* = 0.1309) than in CSF (AUC: 0.625, *p* = 0.4179), but neither reached statistical significance (Figure 1B). Overall, while GFAP levels trended higher in patients with SCA7, they did not strongly discriminate them from the healthy controls.

### 2.4. NfL Levels in Plasma and CSF Are Elevated in SCA7 Patients and Distinguishes Them from Healthy Controls

Multiple studies across neurodegenerative diseases have shown that NfL concentrations in biofluids show prognostic utility [24,28,29,43,44]. As we performed for GFAP, we measured NfL in our study cohort. We observed a significant increase in both plasma (median of 21.6 pg/mL) and CSF (median of 2615 pg/mL) NfL levels in patients with SCA7 compared to the controls (median plasma: 8.2 pg/mL; median CSF: 414.6 pg/mL; Figure 1C and Table 1). Both plasma (AUC: 0.898, *p* = 0.0059) and CSF (AUC: 1.0, *p* = 0.0012) NfL strongly discriminated patients with SCA7 from the healthy controls (Figure 1D). We also observed higher, although not significant, CSF NfL levels in the three asymptomatic SCA7 individuals (median 2412 pg/mL; Table 1) and showed a high AUC value (AUC: 0.875, *p* = 0.0662), when compared to the controls (Figure S2). Overall, these data suggest that neuronal injury is prevalent in patients with SCA7 and may serve to discriminate them from the controls.

### 2.5. Higher Plasma NfL Levels Are Associated with Worse SARA Scores

**Figure 2:**
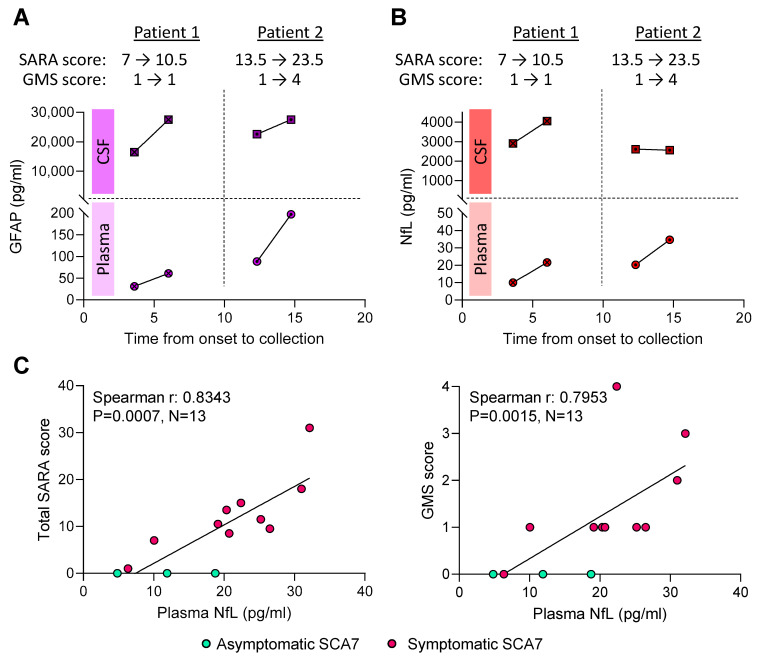
GFAP and NfL levels increase overtime, and higher plasma NfL levels are associated with worse SARA and GMS scores. (**A**,**B**) Plasma and CSF GFAP (**A**) and NfL (**B**) levels were evaluated longitudinally in two symptomatic SCA7 patients. The time in between visits was 29 months for both patients. Progression of their SARA and GMS scores at each visit is included in the figure. (**C**) Spearman correlations were performed to visualize the associations between NfL in plasma or and total SARA (**left**) and GMS (**right**) scores.

We next evaluated whether GFAP or NfL concentrations in biofluids could inform disease progression. Two of our symptomatic SCA7 individuals reported back 29 months later for their follow-up visit, and a second sample of plasma and CSF was collected. Both patients showed worsening of symptoms, as reflected by higher SARA and unchanged GMS scores for Patient 1 and higher SARA and GMS scores for Patient 2, who was utilizing a wheelchair at follow-up (Figure 2A,B). Both GFAP (Figure 2A) and NfL (Figure 2B) levels in plasma and CSF increased with time in both patients. Moreover, Patient 2, who showed worse clinical scores than Patient 1 at the time of sample collection, also had overall higher plasma GFAP and NfL levels (Figure 2A,B). However, neither plasma nor CSF GFAP levels were associated with SARA or GMS scores (Figure S3 and Table 2). In contrast, plasma NfL was significantly associated with worse SARA scores (Spearman r: 0.8343, *p* = 0.0007, N = 13; Figure 2C and Table 2), even when performing linear regression analyses (β: 0.021, 95% CI: 0.008–0.035, *p* = 0.0048; Table 2), but trends were only observed when adjusting for sex, age, and *ATXN7* CAG repeat length (β: 0.013, 95% CI: −0.003–0.029, *p* = 0.0932; Table 2). Similarly, GMS scores were associated with higher plasma NfL levels in the unadjusted analyses (Spearman r: 0.7953, *p* = 0.0015, N = 13, Figure 2C and Table 2), but significance was lost when adjusting for age, sex, and *ATXN7* CAG repeat length (β: 0.074, 95% CI: −0.0042–0.191, *p* = 0.1784; Table 2). CSF NfL levels were not associated with either SARA or GMS scores (Figure S4 and Table 2). Overall, these data suggest that NfL levels in plasma may aid to inform disease progression in SCA7.

## 3. Discussion

SCA7 is characterized by the presence of a polyQ expansion in the ATXN7 protein, leading to changes in protein conformation and the formation of insoluble aggregates [45,46]. These polyQ ATXN7 aggregates interfere with different cellular processes, such as transcription, protein degradation, and glutamate transport [47]. These aggregates accumulate in the nuclear compartment of the cell and are expressed diffusely throughout the brain and various other tissues. These aggregates are thought to be toxic, leading to neuronal loss and consequent symptom development. However, the exact point at which aggregate accumulation becomes toxic remains unclear [45]. Pre-symptomatic *ATXN7* mutation carriers are thus burdened with the notion of when to seek and initiate therapy to halt or prevent disease progression and maintain quality of life. This poses a major challenge for clinicians. Efforts to discover biomarkers for neurodegenerative conditions are critical to improve disease prognosis. Scientific studies aimed at uncovering molecular biomarkers and quantitatively assessing molecular indicators of disease progression are needed. Thus, we aimed to evaluate the utility of GFAP and NfL levels as biomarkers for SCA7, building on their established roles in other neurodegenerative diseases.

GFAP, a neurofilament protein expressed in astrocytes, is released in response to neuronal injury [21] and has been associated with 16 different neurological maladies [48]. GFAP concentrations in biofluids have been evaluated in SCA3, SCA1, and SCA6 with no remarkable findings [35,49,50], although increased GFAP levels were found in plasma from Friedreich’s ataxia patients [51]. Interestingly, no reports to date have investigated GFAP in patients with SCA7. We observed trends of higher GFAP levels in both plasma and CSF in patients with SCA7, along with elevated AUC values (Figure 1B) when compared to the controls. Although these results were not statistically significant, the increase in plasma GFAP in patients with SCA7 contrasts with patterns seen in other SCAs [35,49,50]. Therefore, the contribution of GFAP to SCA pathogenesis could potentially be unique to SCA7 disease pathology. Furthermore, our analysis did not reveal associations between GFAP levels and the clinical characteristics of disease progression and severity. This lack of association may largely be attributed to the small cohort size. It is conceivable that, with a larger cohort, a higher level of distinction could be elucidated.

NfL, a core constituent of axonal cytoskeleton proteins [52], has been established as a marker of axonal damage expressed solely in neurons. It provides an excellent candidate for disease prognostication and aid in assessing therapeutic efficacy [53,54]. It has been extensively investigated as a biomarker in multiple brain-related diseases, such as amyotrophic lateral sclerosis, frontotemporal dementia, Alzheimer’s disease, stroke, multiple sclerosis, and even following COVID-19 infection [29,54,55,56,57,58,59,60,61,62]. Therefore, it is not specific to a single condition. Our group previously found elevated NfL levels in plasma and CSF from patients with SCA3 but not in other SCAs [34]. Studies examining the time-dependent elevation of NfL in plasma and CSF during the disease course of various neurological conditions have shown its peek during the acute phase of the disease [56,58,63]. This suggests its potential utility in determining disease onset among asymptomatic *ATXN7* mutation carriers. In this study, we observed increased NfL levels in both plasma and CSF from two patients with SCA7 from their initial visit to a 29-month follow-up, and three *ATXN7* mutation carriers show high CSF NfL levels. Interestingly, plasma NfL levels increased overtime and significantly correlated with worse SARA scores, indicating its potential to inform on disease progression. The lack of association between CSF NfL and clinical scores is unclear. It is possible NfL rises in the CSF during the early stages of the disease and is then released into the bloodstream at later stages. However, our limited cohort of pre-symptomatic SCA7 cases or SCA7 patients with longitudinal collections restricts our ability to draw definitive conclusions. It is conceivable that CSF may prove to be a superior medium for identifying biomarkers that distinguish different disease states. Furthermore, NfL has been shown to be responsive to therapy. For example, in multiple sclerosis, NfL levels peek during severe relapsing episodes (as characteristic of the disease) and can be lowered following medication administration [63]. Therefore, developing therapies aimed at reducing NfL levels in SCA7 could potentially prolong the pre-ataxic stage and delay disease onset. This approach may also pave the way for future therapeutic trials.

The significance of NfL as a biomarker for SCA7 has just recently been highlighted. In a recent study, increased NfL levels were observed in 15 individuals with SCA7 [36,37,38], and our cohort of 13 *ATXN7* mutation carriers validate such findings. Overall, our cohort showed slightly higher plasma NfL levels (mean: 21.38 pg/mL vs. 15.53 pg/mL [38]). However, previous studies did not include CSF, and we observed higher NfL levels compared to plasma (median SCA7 plasma: 21.6 pg/mL vs. median SCA7 CSF: 2615 pg/mL). More importantly, while previous studies showed increased SARA scores at 1-year follow-up concomitant with decreases in volume loss, they were not able to detect longitudinal changes in NfL [38]. This suggests that CSF may be more sensitive to alterations and could potentially detect changes earlier than in blood. In fact, previous evidence suggests that CSF measurements offer greater sensitivity for detecting NfL than blood [64]. Additionally, increased NfL levels have been observed in CSF, but not in blood, during preclinical stages of other neurodegenerative diseases [65]. Importantly, CSF NfL may provide a more direct measure of central nervous system neuroaxonal injury, whereas blood NfL measurements can be affected by other factors such as kidney function [66], body mass index, and blood volumes [67], among others.

Our study shows increases in both GFAP and NfL at 29 months following first visit for two study participants, where SARA scores increased at a rate of 1.4 (Patient 1) or 4.1 (Patient 2) points/year. Our more significant findings may be explained by the more advanced clinical state of our patients at the time of sample collection, as demonstrated by an earlier median age of onset (30 vs. 36 years [38]) and a lower proportion of pre-ataxic/asymptomatic SCA7 individuals in our cohort (N = 3/13), compared to a previous study (N = 9/15 [38]). Fundamentally, we observed a strong correlation between plasma NfL levels and SARA scores, suggesting that NfL may be indicative of disease progression. Our studies, along with previous findings [38], emphasize the importance of measuring NfL to better understand disease progression and enhance the effectiveness of clinical trials for SCA7. A limitation of our studies stems from the rarity of this condition globally and the challenges associated with collecting human biofluids such as CSF. Nonetheless, we present the largest cohort of symptomatic SCA7 in biomarker studies reported to date, and further support NfL, and potentially GFAP, as biomarkers to inform on disease status, progression, and clinical trial efficacy in SCA7.

## 4. Materials and Methods

### 4.1. Standard Protocol Approvals, Registrations, and Patient Consents

Informed consent was obtained according to standard protocols approved by the Mayo Clinic Institutional Review Board (IRB) ethics committee (IRB 16-009414) and Mayo Clinic IRB approved material transfer agreement between Mayo Clinic and the collaborative institution in Poland. Patients were evaluated by movement disorder specialists (Z.K.W., J.D., J.S.) at Mayo Clinic Florida and Medical University of Gdansk. SCA7 participants originated from Mexico, Poland, Spain, and the United States. The healthy control participants originated from Brazil, Mexico, and the United States, and were confirmed to be negative for the *ATXN7* CAG repeat expansion. All study participants underwent neurological evaluations for the assessment and rating of ataxia (SARA) [40] and gait mobility scale (GMS) [34].

### 4.2. Sample Collection and Preparation

We collected whole-blood, CSF, and/or plasma samples from 3 asymptomatic *ATXN7* mutation carriers and 10 symptomatic SCA7 patients between 2020 and 2023 and from 8 age- and sex-matched healthy controls from 2018 to 2022 (Table 1). Demographic information, as well as clinical data, was recorded for every participant at each visit (i.e., age at sample collection, age of symptom onset, and SARA and GMS scores). Blood and plasma samples were obtained from all 21 participants, and CSF samples were collected from 18 participants. All samples were collected according to standard protocols and procedures at Mayo Clinic Florida and Medical University of Gdansk. Samples were placed on ice immediately following collection and processed within 30 min. Blood and plasma samples were collected during the same visits as the lumbar punctures, where CSF was collected into polypropylene tubes. Samples were centrifuged at 4 °C for 15 min at two different speeds depending on the sample type: 2465 *g* (plasma) and 453 g (CSF). The supernatant was then aliquoted into 200 µL volumes and stored at −80 °C. Blood samples were divided into 2 mL aliquots for DNA extraction.

### 4.3. GFAP and NfL Immunoassays

Both GFAP and NfL were measured on the Simoa^®^ HD-1 analyzer using the Simoa^®^ HD-1 Human Neurology 2-Plex immunoassay for GFAP and NfL (Quanterix, Billerica, MA, USA). Assays were performed by trained personnel. Plasma samples were run at a 4x dilution and CSF was diluted 1:40, as recommended by the manufacturer. Each measurement was performed in duplicate by a technician that was blinded to the identity of the samples. The mean of both measurements was provided along with the percent coefficient of variation (%CV). All measurements showed a CV ≤ 20%.

### 4.4. Genomic DNA Extraction

DNA was extracted from whole blood using the Gentra Puregene Kit (Qiagen, Germantown, MD, USA), according to manufacturer’s instructions, with some modifications. All steps were performed at room temperature. Briefly, 2 mL of whole blood was combined with 6 mL of RBC solution in 15 mL conical bottom centrifuge tubes, incubated for 10 min, and centrifuged at 2000 *g* for 5 min. The supernatant was discarded, and the resulting pellet was resuspended and lysed with 2 mL of Cell Lysis solution, mixed gently, and incubated on a rocking or rotating platform for five minutes or until the solution was completely homogenized. Then, 0.7 mL of Protein Precipitation solution was added, vortexed for 20 s, and centrifuged at 2000 *g* for 5 min. The resulting supernatant was transferred to a new tube containing 3 mL of ice-cold 100% isopropanol and 30 μL of 3 M sodium acetate pH 5.2 (Teknova, Hollister, CA, USA). Next, the sample was mixed by gently inverting the tube 25–50 times until strands or clumps of DNA were revealed. Tubes were incubated 20 min at room temperature or overnight at −20 °C. Samples were centrifuged for 3 min at 2000 *g*, and the supernatant was carefully discarded. Using 0.5 mL of 70% ethanol, the cell pellet was transferred to a DNase RNase free 1.5 mL tube containing 0.7 mL of 70% ethanol and centrifuged at 13,000 *g* for 5 min. Subsequently, the supernatant was discarded, and the resulting DNA pellet was allowed to air-dry by inverting the tube or turning it sideways for no more than 15 min. DNA was finally resuspended in 50–200 μL (based on the size of the pellet) Tris low EDTA suspension buffer (10mM Tris-HCL, 0.1mM EDTA, pH 8.0; Teknova, Hollister, CA, USA), followed by incubation at 65 °C for 10–90 min on a Bioshake (QINSTRUMENTS, Jena, Germany) at 1000 rpm followed by 1–2 h at 55 °C. DNA was stored when three consistent concentrations, measured by Nanodrop, were observed and 260/230 was ≥2.0.

### 4.5. Repeat Expansion Assessment Using a Research Lab-Based Assay from Genomic DNA

CAG repeat expansion genotypic analysis in the *ATXN7* gene was performed for all individuals (mutation carriers and the healthy controls). To assess the length of the CAG repeat expansion, genomic DNA from the patients and controls was amplified using Apex Taq DNA polymerase (Genesee, Rochester, NY, USA) and the following primers: 5′-/FAM/TCGGAGCGGGCCGCGGATGAC-3′ and 5′-CACGACTGTCCCAGCATCACTT-3′ [68]. A touchdown PCR program (3 min 95 °C; 10 cycles of 15 s 95 °C, 15 s 60 °C decreasing 1 °C per cycle, 15 s 72 °C; 25 cycles 15 s 95 °C, 15 s 50 °C, 15 s 72 °C; and 10 min 72 °C; 4 °C) was used. PCR products were diluted, combined with GeneScan 500 LIZ dye Size Standard (Fisher, Plano, TX, USA), and analyzed for size on the ABI 3730 Genetic Analyzer. The GeneMapper™ 4.0 Software (Applied Biosystems, Foster City, CA, USA) was used to determine the allelic repeat size.

### 4.6. Statistical Analyses

Statistical analyses were performed using GraphPad Prism 9.5.0. Differences between study groups were assessed by non-parametric Kruskal–Wallis test, followed by Dunn’s multiple comparison tests. To illustrate and evaluate the associations between NfL or GFAP concentrations and clinical scores, we performed Spearman correlations. Multilinear regression models were also used to evaluate the associations of each biomarker with clinical scores after logarithmically transforming the biomarker data, where confounding variables were included in the adjusted models to account for the potential effects of age, sex, and *ATXN7* CAG repeat length, as indicated. To further determine whether GFAP or NfL can discriminate between the different study groups in our cohort, we performed receiver operator characteristic curves to generate the area under the curve (AUC). An AUC of 0.5 corresponds to a predictive ability equal to that of chance, while an AUC of 1.0 represents a perfect predictive ability. *p* values were considered significant when <0.05 (Kruskal–Wallis, Spearman, AUC analyses) or <0.025 (multilinear regression analyses).

## Figures and Tables

**Table 1:** Characteristics of the study cohort.

Variable	Healthy Controls (N = 8)	Asymptomatic SCA7 (N = 3)	Symptomatic SCA7 (N = 10 Plasma, N = 7 CSF)
Age at ataxia onset (years)	N/A	N/A	30 (7, 55)
Sex (female)	5 (62.50%)	2 (66.67%)	5 (50.00.%)
Age at plasma collection (years)	48.09 (31.74, 62.13)	37.75 (23.87, 54.52)	39.72 (10.12, 66.85)
Age at CSF collection (years)	48.09 (31.74, 62.13)	37.75 (23.87, 54.52)	40.61 (32.61, 65.32)
Disease duration of ataxia at plasma collection (years)	N/A	N/A	8.73 (3.12, 18.82)
Disease duration of ataxia at CSF collection (years)	N/A	N/A	8.61 (3.61, 17.54)
*ATXN7* CAG-repeat length	5.0 (5, 8)	31 (30, 34)	39 (34, 43)
GMS			
0 = Asymptomatic	8 (100.0%)	3 (100.0%)	1 (10.0%)
1 = Impaired gait (no assistance)	0 (0.0%)	0 (0.0%)	6 (60.0%)
2 = Impaired gait (require cane)	0 (0.0%)	0 (0.0%)	1 (10.0%)
3 = Requires a walker	0 (0.0%)	0 (0.0%)	1 (10.0%)
4 = Wheelchair bound	0 (0.0%)	0 (0.0%)	1 (10.0%)
5 = Bedridden	0 (0.0%)	0 (0.0%)	0 (0.0%)
Total SARA	0 (0, 1)	0 (0, 0)	11.5 (3.0, 31.0)
Plasma GFAP (pg/mL)	48.0 (31.8, 108.0)	62.4 (36.6, 71.0)	82.7 (31.2, 242.9)
CSF GFAP (pg/mL)	8906 (4537, 16,433)	14,881 (8888, 32,098)	9318 (5093, 22,573)
Plasma NfL (pg/mL)	8.2 (3.8, 17.7)	11.9 (4.8, 18.7)	21.6 (6.3, 32.2)
CSF NfL (pg/mL)	414.6 (223.5, 769.3)	2412.0 (458.0, 3942.0)	2615.0 (1814.5, 2911.0)

The sample median (minimum, maximum) is provided for continuous variables, and the number (%) of subjects is provided for categorical variables. *ATXN7*: ataxin 7; CAG: cytosine–adenine–guanine; CSF: cerebrospinal fluid; GMS: gait mobility scale; SARA: scale for assessment and rating of ataxia; SCA7: spinocerebellar ataxia type 7. For two cases with a second plasma collection, only information for the first visit was used. N/A: not applicable.

**Table 2:** Associations of GFAP and NfL in plasma and CSF with SARA and GMS scores in *ATXN7* mutation carriers.

		Unadjusted Analysis		Multilinear Regression Model	
Variable	N	β (95% CI)	*p*-Value	β (95% CI)	*p*-Value
Association with plasma GFAP					
SARA	13	0.010 (−0.007, 0.027)	0.2067	0.006 (−0.017, 0.029)	0.5691
GMS	13	0.051 (−0.078, 0.180)	0.4014	−0.002 (−0.162, 0.158)	0.9782
Association with CSF GFAP					
SARA	10	−0.011 (−0.044, 0.021)	0.4398	−0.031 (−0.082, 0.021)	0.1892
GMS	10	0.001 (−0.185, 0.185)	0.9893	0.069 (−0.225, 0.363)	0.5744
Association with plasma NfL					
SARA	13	0.021 (0.008, 0.035)	0.0048	0.013 (−0.003, 0.029)	0.0932
GMS	13	0.129 (0.016, 0.241)	0.0288	0.074 (−0.042, 0.191)	0.1784
Association with CSF NfL					
SARA	10	0.012 (−0.019, 0.042)	0.4026	−0.022 (−0.051, 0.007)	0.1139
GMS	10	0.058 (−0.109, 0.226)	0.4465	0.004 (−0.182, 0.190)	0.9576

β: regression coefficient; CI: confidence intervals. β values, 95% CIs, and *p* values result from unadjusted linear regression models or multilinear regression models adjusted for age, sex, and ATXN7 CAG repeat length, where GFAP and NfL levels were considered on the base 10 logarithmic scale. β values are interpreted as the change in mean GFAP or NfL levels in ATXN7 mutation carriers. *p*-values < 0.025 are considered statistically significant.

## Data Availability

All the data pertinent in this study are included in the figures, tables, and Supplementary Material.

## Supplementary Materials

The following supporting information can be downloaded at: https://www.mdpi.com/article/10.3390/ijms26115070/s1.

## Abbreviations

The following abbreviations are used in this manuscript:

AUC	Area under the curve
ATXN7	Ataxin 7
CAG	Cytosine–adenine–guanine
CV	Coefficient of variation
CSF	Cerebrospinal fluid
GMS	Gait mobility scale
GFAP	Glial fibrillary acidic protein
IRB	Institutional Review Board
NfL	Neurofilament light
SARA	Scale for the assessment and rating of ataxia
STAGA	SPT3-TAFII31-GCN5L acetylase
SCA7	Spinocerebellar ataxia type 7
