# Supplementary material for: Evaluating Glial Fibrillary Acidic Protein and Neurofilament Light as Potential Biomarkers for Spinocerebellar Ataxia 7
Source: Int J Mol Sci. 2025 May 24;26(11):5070. doi: 10.3390/ijms26115070 (PMC12154444; doi:10.3390/ijms26115070)
Supplement: Supplementary file 1 [file ijms-26-05070-s001.zip › ijms-3556308-supplementary.pdf]

**Figure S1.**

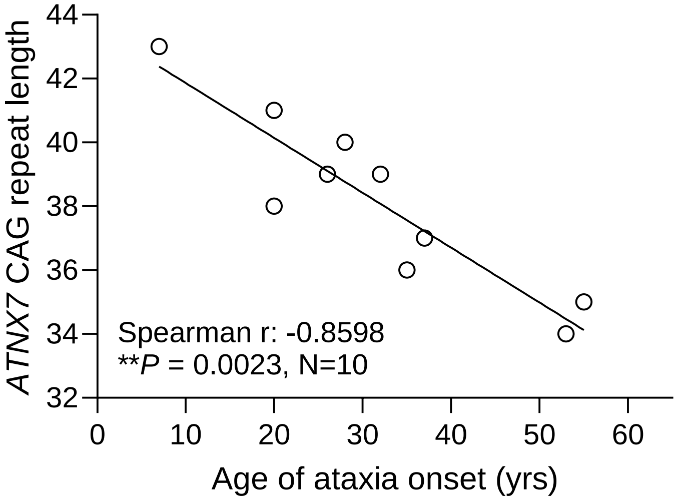


**Figure S1. Age of SCA7 symptom onset negatively correlates with CAG repeat expansion in the *ATXN7* gene.** A Spearman correlation was performed to evaluate the associations between the age of ataxia onset in SCA7 and *AXTN7* CAG repeat length.

**Table S1. *ATXN7* CAG repeat length by study participant from clinically approved and research lab-based assays.**

| Participant | Clinical-based assays (Allele 1/2) | Research lab-based assays (Allele 1/2) |
|---|---|---|
| Control 1 | NA | 5/4 |
| Control 2 | NA | 5/4 |
| Control 3 | NA | 7/7 |
| Control 4 | NA | 5/4 |
| Control 5 | NA | 5/5 |
| Control 6 | NA | 8/5 |
| Control 7 | 5/5 | 5/4 |
| Control 8 | NA | 5/4 |
| Presymptomatic 1 | 40/10 | 34/5 |
| Presymptomatic 2 | 37/NA | 30/5 |
| Presymptomatic 3 | NA | 31/5 |
| Symptomatic 1 | NA | 37/5 |
| Symptomatic 2 | 42/10 | 34/5 |
| Symptomatic 3 | NA | 38/4 |
| Symptomatic 4 | 44/11 | 35/5 |
| Symptomatic 5 | 50/NA | 43/5 |
| Symptomatic 6 | 41/5 | 41/5 |
| Symptomatic 7 | NA | 39/5 |
| Symptomatic 8 | NA | 39/5 |
| Symptomatic 9 | 44/10 | 36/5 |
| Symptomatic 10 | 37/NA | 40/5 |

NA: not available

**Figure S2.**

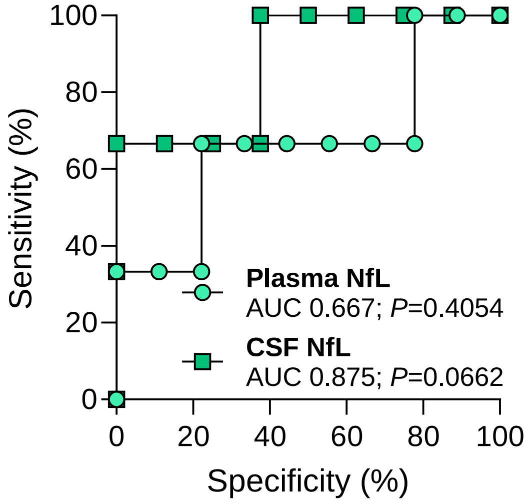


**Figure S2. NfL levels may distinguish asymptomatic SCA7 cases from controls.** Area under the receiver operating curve (AUC) for NfL in plasma (circles) and CSF (squares) of asymptomatic SCA7 individuals (N=3) compared to healthy controls (N=8).

**Figure S3**

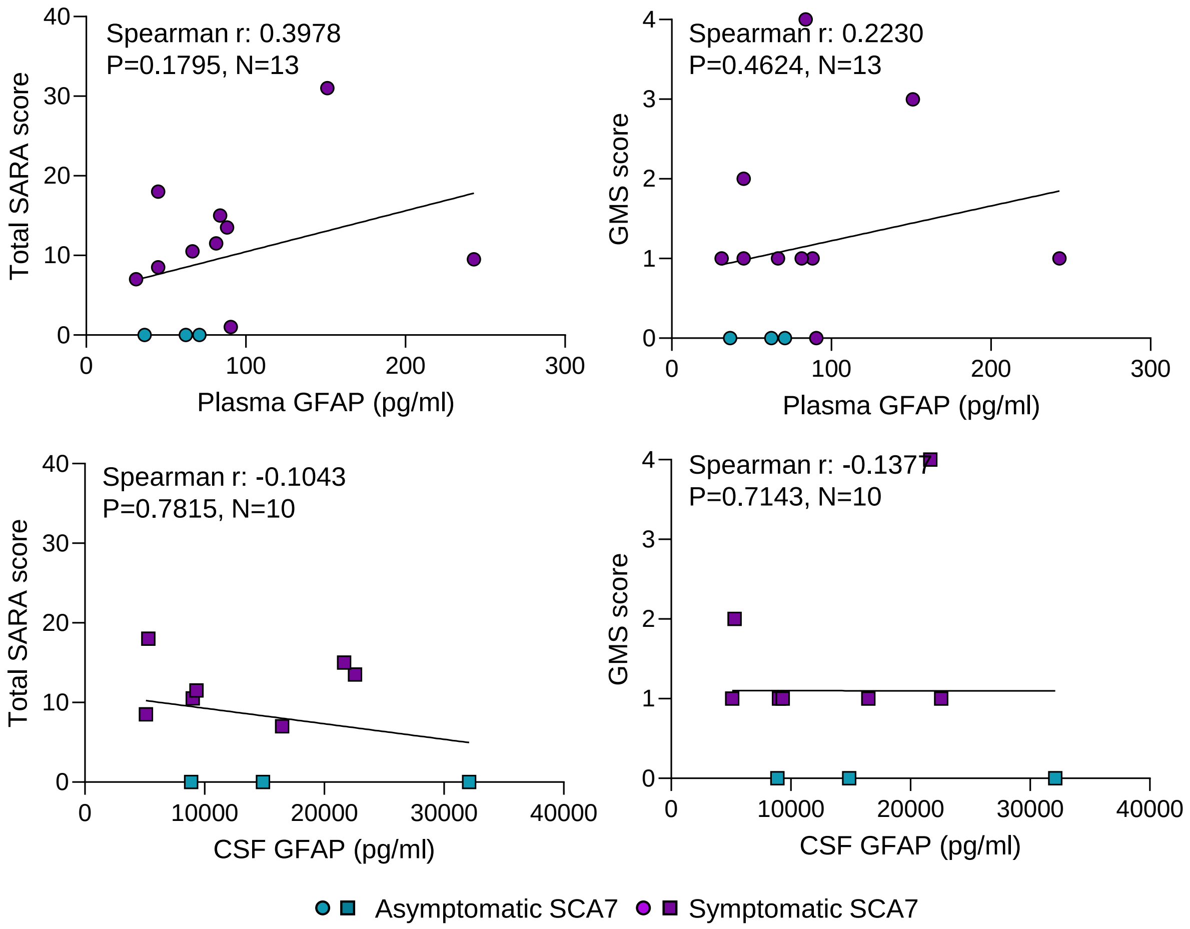


**Figure S3. GFAP levels do not associate with SARA or GMS scores in either plasma or CSF.** Spearman correlations were performed to evaluate the associations between GFAP in plasma or CSF and total SARA and GMS scores.

**Figure S4**

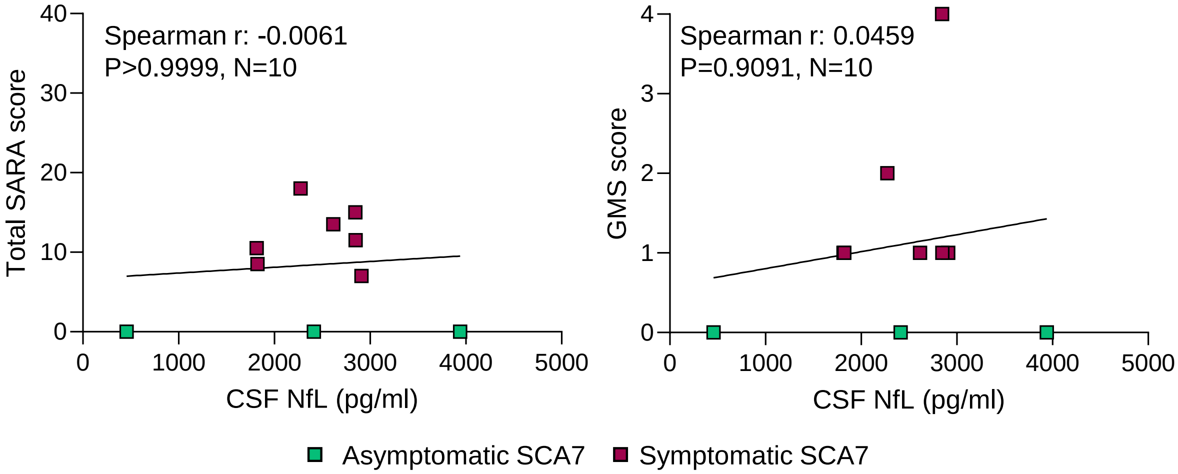


**Figure S4. CSF NfL levels do not associate with SARA or GMS scores.** Spearman correlations were performed to evaluate the associations between NfL in CSF and total SARA and GMS scores.
